# Cyanophage Propagation in the Freshwater Cyanobacterium *Phormidium* Is Constrained by Phosphorus Limitation and Enhanced by Elevated *p*CO_2_

**DOI:** 10.3389/fmicb.2019.00617

**Published:** 2019-03-29

**Authors:** Kai Cheng, Thijs Frenken, Corina P. D. Brussaard, Dedmer B. Van de Waal

**Affiliations:** ^1^Hubei Key Laboratory of Ecological Restoration for River-Lakes and Algal Utilization, College of Resources and Environmental Engineering, Hubei University of Technology, Wuhan, China; ^2^Department of Aquatic Ecology, Netherlands Institute of Ecology, Wageningen, Netherlands; ^3^Department of Marine Microbiology and Biogeochemistry, Royal Netherlands Institute for Sea Research and University of Utrecht, Texel, Netherlands

**Keywords:** climate change, pathogen, cyanobacterial virus, adsorption, one step growth curve, EOP, abortive infection, freshwater

## Abstract

Intensification of human activities has led to changes in the availabilities of CO_2_ and nutrients in freshwater ecosystems, which may greatly alter the physiological status of phytoplankton. Viruses require hosts for their reproduction and shifts in phytoplankton host physiology through global environmental change may thus affect viral infections as well. Various studies have investigated the impacts of single environmental factors on phytoplankton virus propagation, yet little is known about the impacts of multiple factors, particularly in freshwater systems. We therefore tested the combined effects of phosphorus limitation and elevated *p*CO_2_ on the propagation of a cyanophage infecting a freshwater cyanobacterium. To this end, we cultured *Phormidium* in P-limited chemostats under ambient (400 μatm) and elevated (800 μatm) *p*CO_2_ at growth rates of 0.6, 0.3, and 0.05 d^-1^. Host C:P ratios generally increased with strengthened P-limitation and with elevated *p*CO_2_. Upon host steady state conditions, virus growth characteristics were obtained in separate infection assays where hosts were infected by the double-stranded DNA cyanophage PP. Severe P-limitation (host growth 0.05 d^-1^) led to a 85% decrease in cyanophage production rate and a 73% decrease in burst size compared to the 0.6 d^-1^ grown P-limited cultures. Elevated *p*CO_2_ induced a 96% increase in cyanophage production rate and a 57% increase in burst size, as well as an 85% shorter latent period as compared to ambient *p*CO_2_ at the different host growth rates. In addition, elevated *p*CO_2_ caused a decrease in the plaquing efficiency and an increase in the abortion percentage for the 0.05 d^-1^ P-limited treatment, while the plaquing efficiency increased for the 0.6 d^-1^ P-limited cultures. Together, our results demonstrate interactive effects of elevated *p*CO_2_ and P-limitation on cyanophage propagation, and show that viral propagation is generally constrained by P-limitation but enhanced with elevated *p*CO_2_. Our findings indicate that global change will likely have a severe impact on virus growth characteristics and thereby on the control of cyanobacterial hosts in freshwater ecosystems.

## Introduction

Phytoplankton plays a key role in the structure and functioning of aquatic ecosystems. They contribute to approximately half of the biosphere’s net primary production and CO_2_ fixation (Field et al., 1998). Since the industrial revolution, nutrient loading has been progressively increasing, which stimulates phytoplankton growth in many freshwater lakes (Battarbee et al., 2012; Schindler, 2012). Particularly, eutrophication has been associated with the development of harmful cyanobacterial blooms, posing an eminent threat to water quality (O’Neil et al., 2012; Paerl and Otten, 2013). *Phormidium* is a globally widespread genus of filamentous cyanobacteria, distributed from oligotrophic to eutrophic freshwater lakes (Fujimoto et al., 1997; Singh et al., 2014) with increased frequency and intensity over the last decade (McAllister et al., 2016). Although best known for forming benthic mats (McAllister et al., 2016), it can also form planktonic blooms (Srivastava et al., 2015; Iwayama et al., 2017). Various *Phormidium* species are known to produce toxins, causing their proliferations to be a risk for human and ecosystem health (Chaturvedi et al., 2015; Sinang et al., 2015; McAllister et al., 2016; Wood et al., 2017).

As a result of ongoing fossil fuel combustion, atmospheric CO_2_ partial pressure (*p*CO_2_) is predicted to nearly double by the end of this century (Stocker et al., 2013; Friedlingstein et al., 2014). Elevated *p*CO_2_ may affect phytoplankton primary production (Shi et al., 2017), growth rates (Boatman et al., 2017), cell size (Finkel et al., 2010; Mou et al., 2017) and can lead to enhanced cellular carbon to nutrient ratios (Fu et al., 2007; Van de Waal et al., 2010; Garcia et al., 2011). Increased *p*CO_2_, together with other greenhouse gasses, has caused an increase in the global mean temperature and led to warming of the upper water layers of lakes and oceans, which may in turn enhance thermal stratification (Stocker et al., 2013). Subsequently, the supply of nutrients from deeper waters to the surface layer will decrease and thereby suppress primary production (Behrenfeld et al., 2006; Boyce et al., 2011). Freshwater ecosystems, including those dominated by cyanobacteria, may experience nitrogen (N) or phosphorus (P) limitation (Carpenter et al., 1996; Elser et al., 2007; Paerl et al., 2016). Besides a reduced growth rate and biomass build-up (Xu et al., 2010), limitation by nutrients may also lead to an increase in cellular carbon to nutrient stoichiometry (Sterner and Elser, 2002). Such an increase in carbon to nutrient ratios under nutrient limitation can be further enhanced by elevated *p*CO_2_ (Verspagen et al., 2014a), causing a stronger elemental imbalance with potential consequences for higher trophic levels (Sterner and Elser, 2002; Van de Waal et al., 2010).

Population dynamics of cyanobacteria does not only depend on growth related factors such as nutrient availability, but also on mortality related factors like grazers and pathogens. Viruses are highly abundant pathogens and are widely distributed throughout aquatic systems (Suttle, 2007; Wigington et al., 2016). Through lytic infection-induced host lysis, viruses stimulate the microbial loop and are key drivers of nutrient regeneration and element cycling (Gobler et al., 1997; Brussaard et al., 2005a,b; Jover et al., 2014; Mojica et al., 2016). Viruses can strongly control host populations and can be responsible for bloom demise (Brussaard, 2004b; Jenkins and Hayes, 2006; Tijdens et al., 2008; Steenhauer et al., 2016). To date, numerous cyanophages infecting the freshwater cyanobacterium *Phormidium* have been isolated (Safferman and Morris, 1963; Cheng et al., 2007; Liu et al., 2007, 2008), yet little is known about their responses to global change factors.

Viruses rely on their host’s metabolism for reproduction, and their infection success is thus closely linked to the physiological status of the host (Mojica and Brussaard, 2014). Global climate change related alterations in phytoplankton host physiology has been shown to impact virus–host interactions, i.e., viral latent period, burst size and viral infectivity (Wilson et al., 1996; Carreira et al., 2013; Maat and Brussaard, 2016; Maat et al., 2016a,b; Steenhauer et al., 2016). Earlier studies have reported the effects of either elevated *p*CO_2_ or nutrient depletion on the interactions between filamentous cyanobacteria and their cyanophages (Zhou et al., 2015; Shang et al., 2016; Cheng et al., 2017). There are no reports, however, on the combined effects of both factors on filamentous cyanobacterial hosts and their cyanophage infections. Moreover, earlier studies on cyanophages were performed using batch-cultured hosts (Shang et al., 2016), and thus effects could indirectly result from changes in host growth rate and/or growth phase.

To this end, we used chemostats to expose *Phormidium* to a combination of three different P supply rates reaching different extents of P-limitation, both at ambient and elevated *p*CO_2_. In chemostats at steady state, host growth rate is controlled by the dilution rate, and cultures can be kept in a P-limited, rather than P-depleted growth phase. During steady state, subsamples of *Phormidium* from the chemostats were exposed to infection with the cyanophage PP (Cheng et al., 2017). This allowed the assessment of key infection characteristics, such as adsorption, efficiency of plaquing (EOP; the relative proportion of cyanophages forming plaques), abortion percentage (the relative portion of adsorbed cyanophages not forming plaques), latent period, infective production rate (the maximum rate of increase in infections) and infective burst size, in relation to the host response to elevated *p*CO_2_ and P-limitation.

## Materials and Methods

### Experimental Setup

**FIGURE 1:**
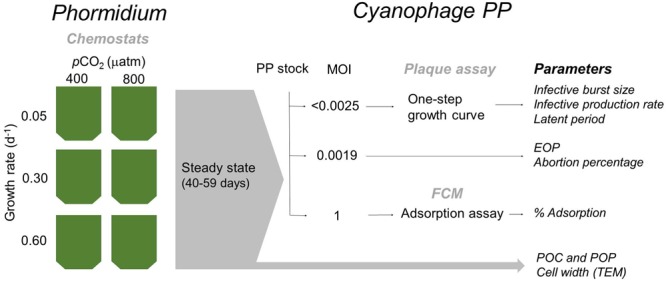
Schematic overview of the experimental setup and sampled parameters. At steady state conditions (after 40–59 days), chemostats were sampled for specific parameters (gray arrow), and for various infection assays (black arrows). MOI, multiplicity of infection; EOP, efficiency of plaquing; FCM, flow cytometry; POC and POP, particulate organic carbon and phosphorus, respectively; TEM, transmission electron microscopy.

A schematic overview of the experiment set-up is provided in Figure 1. In short, the cyanobacterium *Phormidium* was cultured in six chemostats that received a BG-11 medium with reduced PO_4_^3-^ concentrations (i.e., 4.3% of BG-11, see also below) at dilution rates of 0.6, 0.3, and 0.05 d^-1^, at ambient (400 μatm) and elevated (800 μatm) *p*CO_2_. The applied dilution rates resembled 83, 50, and 17% of the maximum growth rate of *Phormidium*. At steady state (when the host cultures were fully conditioned to the different treatments), subsamples were taken to perform triplicate virus infection assays. Steady state was reached at day 40 and experiments lasted until day 59.

### Cultivation of *Phormidium*

*Phormidium* sp. was isolated in 2008 from Donghu lake (i.e., East Lake, Wuhan, China) and was identified by its partial 16S rDNA sequence (Zhou et al., 2010). Cultures (unialgal) were maintained in 100 mL Erlenmeyer flasks at 30–40 μmol photons m^-2^ s^-1^ (14:10 h light:dark cycle) and 24°C. Batch mono-cultures were diluted 1:10 (v:v) weekly using sterile filtered (0.22 μm polyethersulfone membrane filter; Sartopore Midicap; Sartorius A.G., Göttingen, Germany) standard BG-11 medium (Andersen, 2005) to keep the cells exponentially growing (maximum growth rate of about 0.72 d^-1^). Prior to transfer to the chemostats, *Phormidium* was acclimated to BG-11 medium with reduced PO_4_^3-^ concentrations (10 μM K_2_HPO_4_, i.e., 4.3% P of standard BG-11 medium) for at least 14 days (i.e., about 14 generations).

The chemostat cultures consisted of flat panel 1.7 L glass vessels (Huisman et al., 2002) at a light–dark cycle of 16:8 h. The average light intensity was calculated as (*I*_in_ – *I*_out_)/(ln *I*_in_ – ln *I*_out_) (Huisman et al., 2002), where *I*_in_ is the average incoming light (PL-L 24W/840/4p, Philips, Netherlands) intensity, and *I*_out_ was the average outgoing light intensity. The average (non-limiting) light intensity was 68 μmol photons m^-2^ s^-1^ at day 1. The higher irradiance and longer light period compared to pre-culturing was chosen to avoid light limitation during the experiments. Temperature was kept constant at 25 ± 1°C by means of a cooling finger, and aeration ensured homogeneous mixing. Cultures were supplied with compressed air at a fixed flow rate of 20 L h^-1^, with a *p*CO_2_ of 400 μatm in the control conditions and 800 μatm in the elevated *p*CO_2_ conditions. The *p*CO_2_ were confirmed by using an Omniport 30 multifunctional handheld CO_2_ meter (E+E Elektronik GmbH, Engerwitzdorf, Austria).

At steady state during days 40–59, subsamples were taken for particulate organic carbon (POC), particulate organic phosphorus (POP) and dissolved inorganic phosphate analysis. Transmission electron microscopy (TEM) pictures were taken to monitor changes in the morphology of *Phormidium* cells. The dissolved inorganic phosphate concentrations at steady state were below the detection limit (<0.01 μM) for all treatments. For analyses of POC, 10 mL of cell suspension was filtered through a demi-water pre-washed 25 mm GF/F filter (Whatman^TM^, Maidstone, United Kingdom) (Frenken et al., 2016), which was subsequently dried overnight at 60°C and stored in a desiccator. Most of the heterotrophic bacteria (sampled and counted using the protocol by Marie et al. (2001) in combination with a Beckman Coulter MoFlo Legacy Cell Sorter flow cytometer) in the *Phormidium* cultures passed through the nominal 0.7 μm pore-size GF/F filters and so we consider the POC and POP results to mainly reflect the cyanobacterial elemental composition. The filtrate was used to measure the phosphate concentration with a QuAAtro segmented flow analyzer (Seal Analytical Incorporated, Beun de Ronde, Abcoude, Netherlands). For POC analyses, a subsample of 14.5% was taken from each filter by perforation. The acquired punches were then folded in a tin cup and POC was analyzed on a Flash EA 1112 NC analyzer (Interscience, Milan, Italy). POP was determined by first incinerating the remaining 85.5% subsample for 30 min at 500°C, followed by a 2% persulfate digestion step in the autoclave for 30 min at 121°C. Subsequently, the digested samples were analyzed for PO_4_^3-^ using a QuAAtro segmented flow analyzer (Seal Analytical Incorporated, Beun de Ronde, Abcoude, Netherlands). Reported POC, POP and cellular C:P ratios indicate means of the experimental period (*n* = 3), during which all virus infection assays were performed.

To inspect any morphological changes in the cyanobacteria in response to different CO_2_ and P supply rates, TEM photographs were taken for every treatment. To this end, 20 mL of *Phormidium* was concentrated to 0.5 mL by centrifugation at 8,000 × *g*, at 4°C for 90 min on day 59, after which 0.2 mL glutaraldehyde (2.5%) was immediately added to the pellet for fixation. Thereafter, additional fixation was done by immersing the sample in 1% osmic acid for 4 h, after which the sample was concentrated by centrifugation at 3,000 × *g* for 5 min at 4°C and 0.5 mL PBS was added to the precipitate before washing. Centrifugation and washing were repeated three times. Dehydration was done by immersing the sample in alcohol solutions from 50 to 100% by a gradual increasing gradient with steps of 10%. Samples were then embedded in Spurr resin (ERL-4206). Ultra-thin sections were made by a UC7 ultramicrotome (Leica, Germany) and stained with uranyl acetate and lead citrate. A Tecnai G20 TWIN TEM (FEI, United States) was used to measure the cyanobacterial cell width at a magnification of 1700× to 5000×.

### Culturing of Cyanophage PP

The cyanophage PP, named after the respective first letter of its two known hosts (*Plectonema boryanum* IU 594 and *Phormidium foveolarum* IU 427), was isolated in 2001 from a eutrophic freshwater pond in Wuhan, China (Zhao et al., 2002; Cheng et al., 2007). It is characterized as a short-tailed, icosahedral-shaped, double-stranded DNA virus (Cheng et al., 2007). To prepare the PP stock [with a titer of 1.07 × 10^8^ plaque forming units (PFU) mL^-1^] for the infection assays, 10 mL stored PP lysate was inoculated with 100 mL batch cultured *Phormidium* at exponential growth, using a multiplicity of infection (MOI, defined as the ratio of the titer of cyanophage PP to the cell density of *Phormidium*) of 1. The mixture was cultured in the same condition as the batch culture of *Phormidium* for 2 days. Subsequently, for the preservation of cyanophage stock, 11 mL chloroform was added to the mixture followed by rigorous manual shaking for 1 min (Fox et al., 1976). Then the mixture was placed at 4°C without shaking for 1 h before 20 mL of 100 mL supernatant was carefully pipetted out (Fox et al., 1976) and stored at 4°C (Cheng et al., 2007). This PP stock was diluted for more than 100 times for the infection assays. To assess the titer of this stock, it was serial diluted by standard BG-11 medium and a plaque assay was performed (Suttle, 1993) up to 1 week prior to the infection assays. For the plaque assay, 12 mL solid standard BG-11 media with 1% agar (Sigma, A1296, St. Louis, MO, United States) was plated in a 90 mm diameter petri-dish to form the bottom layer. Then 0.1 mL of serial diluted stock sample was mixed with 1.9 mL of batch cultured *Phormidium* cells (with a density of 2.0–6.0 × 10^7^ cells mL^-1^) in standard BG-11 media. Cultures were rapidly plated in the above mentioned petri-dish with 2 mL pre-heated 65°C standard BG-11 media with 1% agar. The plates were subsequently incubated for 2–3 days under the same culture conditions as *Phormidium* batch cultures, after which plaques were counted.

### Infection Assays

All virus infection assays, i.e., the adsorption assay, viral growth curve, EOP, and abortion percentage assays, were performed during steady state between days 50 and 59 of the chemostat experiments. During this period, culture material for the assays was sampled from the chemostats approximately 3 h after the start of the light period. Average light intensities in the chemostats were 52–58 μmol photons m^-2^ s^-1^ (non-limiting light condition) during the period infection assays were performed (from day 50 to 59). To assess the various cyanophage characteristics, samples were taken for short term (<8 h) infection assays with cyanophage PP, which were all performed in triplicate.

To homogenize *Phormidium* host cultures and to separate filaments, 10 mL of sample was pipetted up and down with 5 mL pipettes for at least 10 times, after which the culture was sieved over a 30 μm nylon mesh. The filtrate was concentrated by centrifugation at 16,000 × *g* for 1 h at 25°C, after which the cyanobacterial pellet was re-suspended in 10 mL low P containing BG-11 medium. Cell abundances were then calculated from trichome length measurements, determined using a hemocytometer (with a volume of 0.1 mm^3^) on an inverted microscope (DMI 4000B, Leica Microsystems CMS GmbH, Mannheim, Germany). First, the average cell length (A) was assessed by dividing the total length of 10 randomly selected trichomes with their cell numbers. Afterward, the total length of all trichomes in at least 10 counting chambers were measured for each sample, and the average total length of trichomes per counting chamber (L) was calculated. The average cell density (per mL) was subsequently calculated as L/A × 10^4^. After counting, all cultures were then further diluted in low P containing medium to equalize host cell density to 1 × 10^6^ cells mL^-1^.

### Adsorption Assay, Efficiency of Plaquing and Abortion Percentage

For the adsorption assay, 4 mL of the diluted *Phormidium* host culture was mixed with the cyanophage PP stock at MOI of 1 and grown at *p*CO_2_ and non-limiting light conditions comparable to the chemostats (i.e., 60 μmol photons m^-2^ s^-1^). Samples of 0.8 mL were taken at different time points (0, 30, and 60 min). Next, these samples were centrifuged at 16,000 × *g* at 4°C for 25 min of which 400 μL supernatant was fixed with 25%-glutaraldehyde (Merck, Darmstadt, Germany) to a final concentration of 0.5%, where after the sample was stored in the dark at 4°C and analyzed within 24 h. Viruses were sampled and enumerated using flow cytometry according to the protocol by Brussaard (2004a) with modification by Mojica et al. (2014). In short, samples were diluted in Tris-EDTA buffer (pH 8.2; Mojica et al., 2014) and stained with SYBR Green I (final concentration of 5 × 10^-5^ of commercial stock, Sigma-Aldrich, St. Louis, MO, United States) for 10 min in the dark at 80°C. Samples were analyzed on a MoFlo Legacy Cell Sorter with a 488 nm argon laser and the trigger on green fluorescence (of the nucleic acid-specific staining by SYBR Green I). The adsorption ratio at any time point *t* was calculated as 100% - (*V_t_*/*V_0_*), where *V_0_* and *V_t_* are the virus abundances at time point 0 and *t*, respectively.

To determine the EOP and the abortion percentage, the cyanophage PP stock was mixed with 4 mL diluted host cell cultures (0.7 × 10^6^ cells mL^-1^) to a sufficient low final concentration reaching 1,300 PFU mL^-1^ (defined as *P_0_*, determined by plaque assay, i.e., at MOI of 0.0019). This low concentration was used to avoid multiple adsorption of phages adsorbed to a single trichome. The mixed samples were then incubated in the light (60 μmol photons m^-2^ s^-1^) for 15 min after which 2 mL was subsampled and centrifuged at 16,000 × *g* at 25°C for 90 min. Then, the titer in both the supernatant and pellet were determined by plaque assays (Suttle, 1993), providing *P_1_* and *P_2_*, respectively. The EOP was calculated as *P_2_*/*P_0_*, and the abortion percentage was calculated as 100% -*P_2_*/(*P_0_* - *P_1_*).

### Latent Period, Production and Infective Burst Size

For the one step growth curve, cyanophage PP stock was added to the above mentioned filtrate at a low MOI of 0.00025–0.0025 (i.e., final concentration of 250–2500 PFU mL^-1^) to avoid multiple infections to a single trichome after a first round of lysis (Cheng et al., 2017). After 10 min of incubation in the light (60 μmol photons m^-2^ s^-1^) to allow adsorption, 4 mL mixtures were centrifuged at 16,000 × *g* for 25 min at 25°C. The pellets were collected, washed twice in low P containing BG-11 medium, and then resuspended in 25 mL of low P containing BG-11 medium and incubated at *p*CO_2_ and light conditions similar to the chemostat conditions. Since the released new cyanophages may rapidly adsorb to the nearby hosts, cultures were shaken on an INFORS Multitron incubator shaker (INFORS HT, Switzerland) for 6 h at 40 rpm to disperse the new cyanophages before they attached to nearby hosts (thereby preventing multiple infections of a single trichome). Subsequently, to make sure 5–150 plaques can be formed in a single plate, 0.1 and 1 mL of subsamples were taken from the resuspended cultures every hour for a period of up to 8 h, and mixed with 1.9 or 1 mL of batch cultured *Phormidium* cells in standard BG-11 medium, respectively (equaling a total of 2 mL). Then, the cyanophage titers of the mixture were determined using the plaque assay method (Suttle, 1993) as mentioned above. To normalize the data, titers at each time point were divided by the titer at *t_0_* to get the relative titers. To determine the latent period, one step growth curves were constructed directly by using those relative titers, and the end of latent period was determined as the time point when the average relative titer increased to higher than 1. To determine the average infective burst size (i.e., the number of new infections released by a single infected host cell) and the infective production rate (i.e., the maximum rate of increase in infections), a modified Gompertz sigmoid growth function (Zwietering et al., 1990) was constructed based on the relative titer:

$$y=B\times \exp\left(-\exp\left(\frac{r_{m}\times e}{B}(\lambda -t)+1\right)\right)+1$$

where *y* indicates the titer at time *t, B* the infective burst size, *r_m_* the infective production rate, *e* the mathematical constant (i.e., 2.718), and *λ* is the point on the *x*-axis where the slope from the maximum increase meets *y* = 1. We note that the infective burst size and infective production rate are based on plaque forming units, and thus represent the maximum number of infections after one lytic cycle and the maximum increase of infections, respectively. Fits were performed using least square fitting with the Microsoft Excel 2013 Solver GRG non-linear fitting procedure with a multiStart population size of 200.

### Statistical Analysis

The virus infection data were Ln- or square-root-transformed to improve normality and equality of variance, which were confirmed using the Kolmogorov–Smirnov test and Levene’s test, respectively. Significance of differences between treatments were tested using an one-way ANOVA, followed by *post hoc* comparison of the means using Fisher’s Least Significant Difference (LSD) test if the data was homoscedastic, or using the Games-Howell test if the data were not homoscedastic. The interaction effect between the CO_2_ treatments and host growth rates was tested using a two-way ANOVA. Correlation analysis was performed by using a Spearman’s test. All statistics were performing with SPSS Statistics 17.0 (IBM Inc., United States).

## Results

**FIGURE 2:**
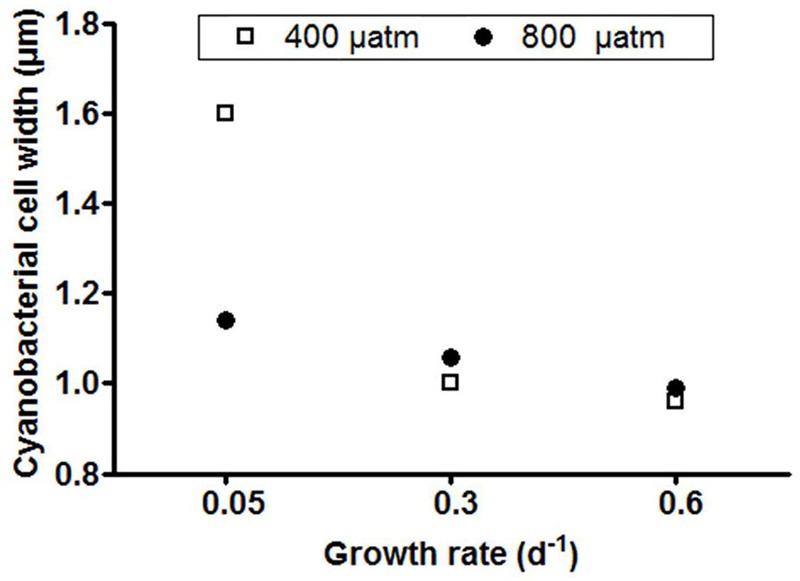
Host cell width for each treatment. Symbols represent means of 20 cell measurements from one thin section for each chemostat.

At steady state, *Phormidium* showed lowest POC concentrations at the highest growth rate (0.6 d^-1^) at both *p*CO_2_ levels (Table 1). Similarly, POP concentrations were lowest at the highest growth rate, and showed a distinct increase with decreasing growth rate both under ambient and elevated *p*CO_2_ (Table 1). Average C:P ratios generally increased with elevated *p*CO_2_ across growth rates, with a strongest effect at the lowest growth rate (Table 1). There was furthermore a significant interaction between growth rate and elevated *p*CO_2_ (*P* < 0.05, Table 2). For the elevated *p*CO_2_ treatment, the average width of the cyanobacterial cells at the lowest growth rate was 8–15% larger than for intermediate and highest growth rate (Figure 2). For the ambient *p*CO_2_ treatment, the cell width was 60–67% higher at the low growth rate as compared to the intermediate and highest growth rates.

**Table 1:** Overview of host biomass and stoichiometry, with particulate organic carbon (POC) and phosphorus (POP) and cellular C:P ratios.

Treatments		POC (μM)		POP (μM)		Cellular C:P	
*p*CO_2_	Growth
(μatm)	rate (d^-1^)	Mean	SD	Mean	SD	Mean	SD
400	0.05	1567	580	9.1	3.8	173	17
800	0.05	2724	258	7.6	1.8	371	88
400	0.3	2189	385	6.7	1.5	329	17
800	0.3	1682	1310	4.3	2.5	361	97
400	0.6	970	221	4.1	0.9	240	34
800	0.6	1170	662	3.7	1.7	305	52

*Values denote mean (±SD, *N* = 3) over the steady state period of the experiment.*

**Table 2:** Overview of the two-way ANOVA results showing impacts of *p*CO_2_, growth rate and their interaction on host cellular C:P ratios, EOP, infective production rate, and infective burst size.

Data	*p*CO_2_			Growth rate			Interaction		
	df	*F*	*P*	df	*F*	*P*	df	*F*	*P*
Cellular C:P ratio	1	16.66	0.002	2	4.60	0.033	2	5.69	0.018
EOP	1	64.46	<0.001	2	32.54	<0.001	2	81.12	<0.001
Infective production	1	4.45	0.057	2	46.86	<0.001	2	7.78	<0.001
rate^a^
Infective burst size	1	20.99	0.001	2	46.25	<0.001	2	17.33	<0.001

*^*a*^Data were Ln-transformed to improve equality of variance.*

**FIGURE 3:**
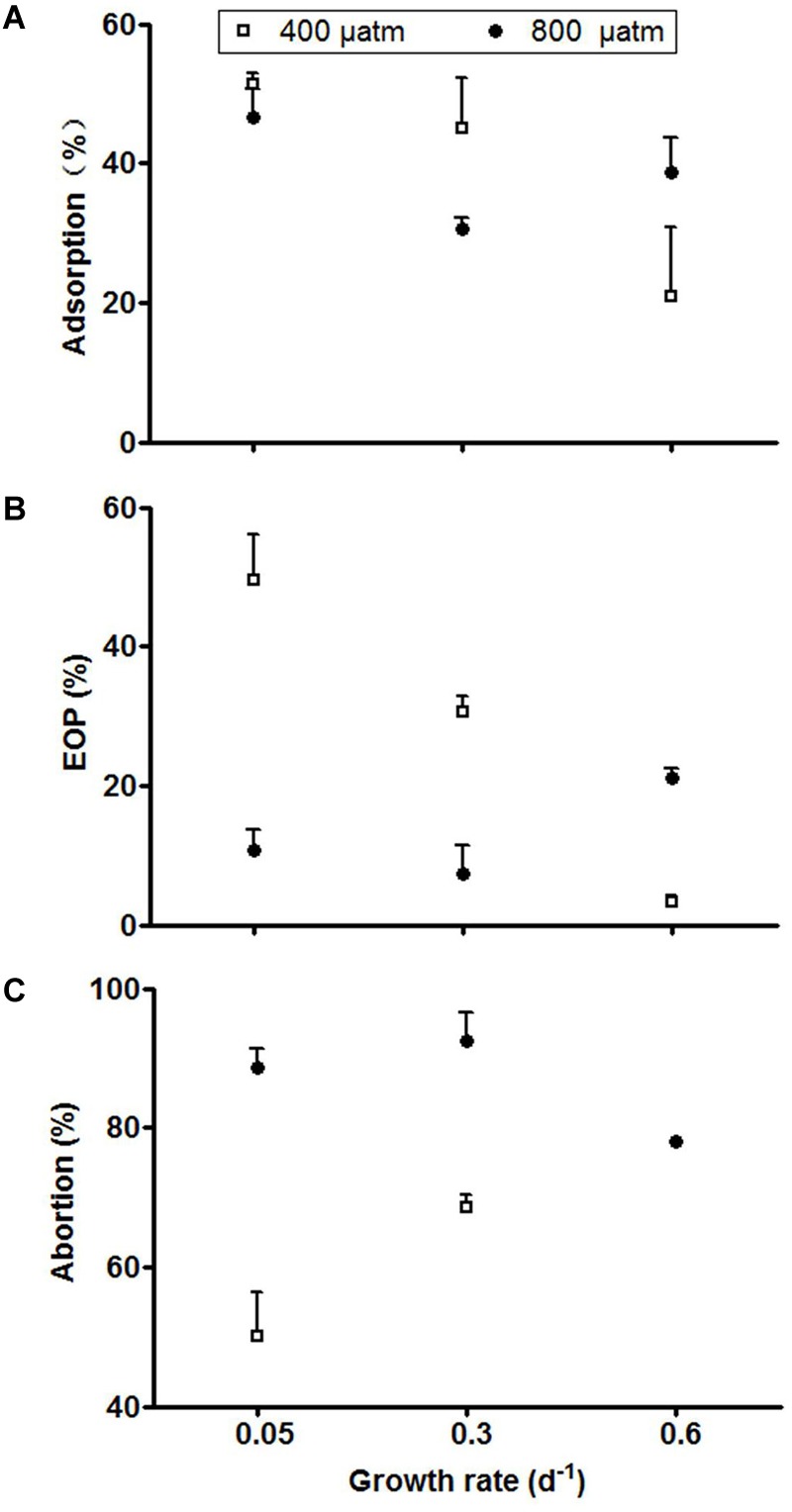
Adsorption **(A)**, EOP **(B)**, and abortion percentage **(C)** of cyanophage PP. Symbols indicate mean ± SD (*n* = 3). The abortive percentage for the highest growth rate under ambient *p*CO_2_ could not be assessed since viral adsorption was not detected.

The infection assays showed clear effects of host growth rate, and thereby the degree of P-limitation, as well as *p*CO_2_ on the various cyanophage infection characteristics (Supplementary Table 1). The adsorption (% viral particles adsorbed to the host cells after 60 min) at ambient *p*CO_2_ doubled with a decrease in growth and thereby an increasing strength of P-limitation (from 0.6 to 0.3 d^-1^; Figure 3A). Under elevated *p*CO_2_ conditions, the relatively low adsorption at high host growth rate (0.6 d^-1^) increased as compared to the ambient *p*CO_2_ conditions. The EOP (% cyanophages that lead to infection) at ambient *p*CO_2_ showed a distinct positive correlation with the extent of P-limitation (Figure 3B). This effect disappeared, however, at elevated *p*CO_2_. At low host growth rate (0.05 d^-1^) the decrease in response to elevated *p*CO_2_ was even 67%. The abortion percentage (% adsorbed cyanophages that did not lead to infection) at ambient *p*CO_2_ was lowest at most severe P-limitation (from 50% at 0.05 d^-1^ as compared to 69% at 0.3 d^-1^; Figure 3C). Under elevated *p*CO_2_, abortion of PP increased strongly for the more severe P-limited treatments (from 50 to 89% at 0.05 d^-1^, and from 69 to 93% at 0.3 d^-1^; *P* < 0.001).

**FIGURE 4:**
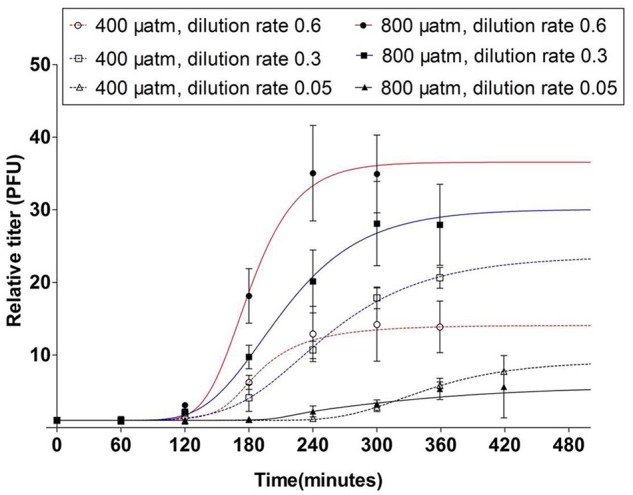
One step growth curves of the cyanophage PP. Symbols indicate mean ± SD (*n* = 3). The relative titers are the cyanophage titers at each time point relative to those at *t_0_*.

The latent period was strongly affected by the extent of P-limitation, i.e., the latent period was prolonged to 180–240 min at severe P-limitation, irrespective of the *p*CO_2_ condition (Figure 4). At the other P-limitation conditions, the latent period was 120–180 min at ambient *p*CO_2_. However, elevated *p*CO_2_ shortened the latent period to 60–120 min (Figure 4).

**FIGURE 5:**
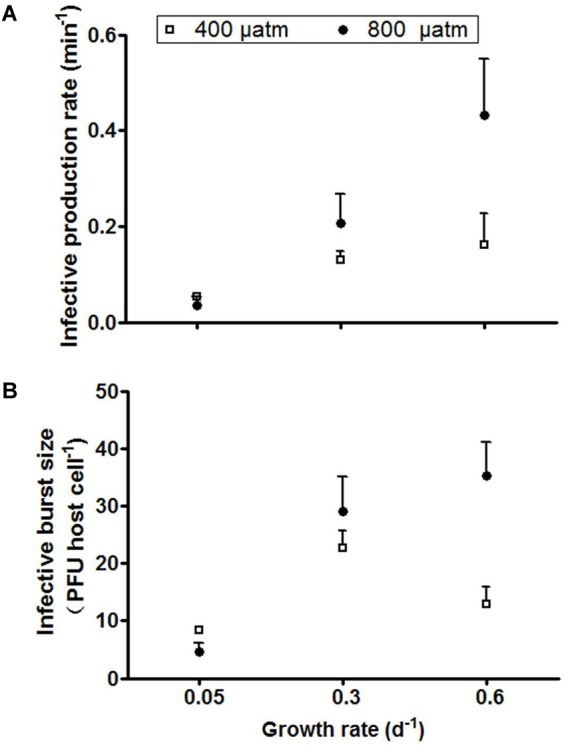
Infective production rate **(A)** and infective burst size **(B)** of cyanophage PP. Symbols indicate mean ± SD (*n* = 3).

The infective production rate at ambient *p*CO_2_ decreased strongest with increasing P-limitation, i.e., it decreased by 62% from host growth rate 0.3 to 0.05 d^-1^ (Figure 5A). Elevated *p*CO_2_ caused a marked increase in infective production rate for the higher growth rate cultures, particularly for the cultures grown at 0.6 d^-1^ (from 0.16 to 0.54 min^-1^). The infective production rate was thus significantly influenced by both *p*CO_2_ and the extent of P-limitation, with both factors showing a clear interactive effect (*P* < 0.001, Table 2).

The trends of infective burst size with P-limitation and with *p*CO_2_ were largely comparable to the infective production rate. The lowest burst size was observed at the lowest growth rate with severe P-limitation (Figure 5B), while elevated *p*CO_2_ caused an increase in burst size at particularly the highest growth rate (from 23 to 35 PFU cell^-1^; Figure 5B). The extent of P-limitation, elevated *p*CO_2,_ as well as their interaction significantly affected virus burst size (*P* < 0.01; Table 2). In other words, the effect of *p*CO_2_ depended on the extent of P-limitation and seemed smallest under severe P-limitation (lowest growth rate) and largest at highest host growth rate (0.6 d^-1^).

## Discussion

### Host Status

The observed C:P ratios of *Phormidium* at steady state were all distinctly higher as compared to the Redfield C:P ratio of 106, indicating that phosphorus limited growth (Table 1). This was furthermore confirmed by the residual phosphate concentrations in the chemostats that were all below detection limit (i.e., <0.01μM). The average cellular C:P ratios of *Phormidium* significantly increased with P-limitation and even more so to *p*CO_2_ (Table 2). Cellular C:P ratios can be indicative for the extent by which cells are P-limited (Geider and LaRoche, 2002; Sterner and Elser, 2002), which is particularly evident for the displayed increase in C:P ratios from a growth rate of 0.6 to 0.3 d^-1^ (from 240 to 329, and 305 to 361, for ambient and elevated *p*CO_2_, respectively). We note that C:P ratios at ambient *p*CO_2_ and a growth rate of 0.05 d^-1^ were distinctly lower than observed in the other treatments (Table 1). This might be associated to the observed increase in cell size, where apparently POP is more strongly accumulated as compared to POC (Figure 2 and Table 1). Irrespective of growth rate, elevated *p*CO_2_ resulted in higher C:P ratios of *Phormidium*. This may be due to continued fueling of the cells by CO_2_, while at the same time the lack of P supply (i.e., through the fixed dilution rates) made the C:P ratio even higher. Similarly, the C:P ratio increased with elevated *p*CO_2_ for *Micromonas pusilla*, and while the effect seemed stronger at lower growth rates (i.e., stronger P-limitation), no significant interaction between elevated *p*CO_2_ and P-limitation was observed (Maat et al., 2014).

### Virus Infection Experiments

Generally, our results show that adsorption increased with the extent of P-limitation. More specifically, when *Phormidium* growth was severely P-limited (0.05 d^-1^, i.e., 17% of its maximum growth rate), adsorption of viruses was 51% as compared to 21% at the highest host growth rate (0.6 d^-1^, i.e., 83% of maximum growth rate). Little is known about the viral resistance mechanism of *Phormidium*, except that a *P. uncinatum* mutant strain was viral resistant due to the complete absence of viral adsorption (Bisen et al., 1986). Generally, adsorption of viruses depends on density (Murray and Jackson, 1992), as well as on host cell size, where larger host cells provide a greater surface area for contact (Hadas et al., 1997). In all adsorption assays, host and cyanophage abundances were kept similar and the observed differences can thus not be explained by changes in contact rate due to host density differences. However, *Phormidium* cells under the most severe P-limitation at 400 *μ*atm *p*CO_2_ were overall 40% wider than in the other treatments, which may have contributed to an increased contact rate (Murray and Jackson, 1992).

Increased adsorption of viruses may also derive from enhanced production of extracellular polymeric substances (EPS) that is often observed under nutrient limitation or depletion (Zhao et al., 2014), as this serves as a sink for excess fixed cellular carbon under unbalanced carbon to nutrient stoichiometry (Otero and Vincenzini, 2004; Boonchai et al., 2015; Gonzalez-Garcia et al., 2015). However, the relatively high EOP and low abortion percentage at severe P-limitation and ambient *p*CO_2_ suggest that putatively higher EPS production does not prevent successful infection. Virus adsorption influences EOP, and the observed changes in adsorption ratio indeed correlated to those observed for EOP (*P* < 0.05). Thus, shifts in EOP could, at least partially, be explained by changes in adsorption ratio, with a higher adsorption leading to a higher EOP. Moreover, EOP increased significantly with elevated *p*CO_2_ (*P* < 0.05) at the highest host growth rate. In contrast, elevated *p*CO_2_ at the lowest and intermediate host growth rates led to a strong decrease in EOP, which was associated to a concomitant increase in abortion percentage (*P* < 0.05). We hypothesize that under severe nutrient limited conditions excess carbon from photosynthesis cannot be allocated to growth because of a lack of nutrients, and may lead to the accumulation of EPS (Boonchai et al., 2015). This may subsequently result in a lower EOP by enhanced host resistance, possibly through trapping the phages in EPS and thereby preventing successful infection (Looijesteijn et al., 2001). Apparently, the potentially extra EPS production at elevated *p*CO_2_ did not affect the adsorption success. We recommend future studies to include EPS measurements to better understand the putative role of EPS in host resistance against virus infections.

P-limitation of *Phormidium* strongly reduced the propagation of infections, with a prolonged latent period, lowest infective production rate and infective burst size at severe P-limitation (0.05 d^-1^). These findings are comparable to other cyanophage and eukaryotic algae virus-host systems (Table 3). This suggests a more general negative effect of low P availability on virus infections, with an increased in latent period and a decrease in burst size with increasing P-limitation or under P depletion. The length of the latent period is mainly determined by synthesis of lysozymes, while burst size is mainly determined by the synthesis of proteins (Hadas et al., 1997). Thus, both factors depend on protein synthesis, for which the efficiency may possibly decrease with P-limitation through reduced cellular RNA content (Hessen et al., 2017). Alternatively, reduced photophosphorylation by the host as a consequence of P-limitation may possibly lead to depletion of energy reserves (Maat et al., 2016a), and as such limit cyanophage protein synthesis (Maat et al., 2016a; Puxty et al., 2018). Also, the observed negative effects of P-limitation on virus infectivity can possibly be explained by the high P demands of the viruses, as indicated by their generally low C:P ratio reflecting relatively high amounts of P-rich nucleic acids (Bratbak et al., 1993; Clasen and Elser, 2007).

**Table 3:** Overview of responses of virus infection characteristics (latent period and burst size) to phytoplankton exposed to nutrient limitation or elevated *p*CO_2_.

Virus	Hosts		Habitat	Nutrient limitation		*p*CO_2_ elevation		Reference
				Latent	Burst	Latent	Burst
	Group	Species		period	size	period	size	
Cyanophage PP	C	*Phormidium* sp.	F	↑	↓	↓	↑	Present work
Cyanophage PP	C	*Phormidium* sp.	F	↑	↓	NA	NA	Shang et al., 2016
Cyanophage PP	C	*Plectonema boryanum* IU597	F	NA	NA	↓	↓	Cheng et al., 2017
Cyanophage PP	C	*Leptolyngbya* sp.	F	NA	NA	–	↑	Zhou et al., 2015
Cyanophage S-PM2	C	*Synechococcus* sp. WH7803	M	–	↓	NA	NA	Wilson et al., 1996
Cyanophage S-PM2	C	*Synechococcus* sp. WH7803	M	NA	NA	↓	↓	Traving et al., 2014
MpV-08T	Ch	*Micromonas pusilla* Lac38	M	↑	↓	–	–	Maat et al., 2014
MpV-08T	Ch	*Micromonas pusilla* Lac38	M	↑	↓	NA	NA	Maat and Brussaard, 2016; Maat et al., 2016a,b
PgV-07T	H	*Phaeocystis globosa* G(A)	M	↑	↓	NA	NA	Maat and Brussaard, 2016; Maat et al., 2016a
PpV01	H	*Phaeocystis pouchetii*	M	NA	NA	–	–	Carreira et al., 2013
EhV-99B1	H	*Emiliania huxleyi* BOF	M	NA	NA	↑	↑	Carreira et al., 2013

*C, cyanobacteria; Ch, chlorophyta; H, haptophyta; F, freshwater; M, marine; ↑, increase; –, no impact; ↓, decrease; NA, not available.*

Elevated *p*CO_2_ led to a shortened latent period of cyanophage PP, an increased infective production rate, and an increase in infective burst size for the intermediate and fastest growing hosts. This enhanced cyanophage proliferation may result from a CO_2_-driven increase in net photosynthesis by the host, as suggested by the higher C:P ratios (Fu et al., 2007; Maat et al., 2017). An increased cyanophage burst size in response to elevated *p*CO_2_ was also observed for the nutrient replete filamentous cyanobacterium *Leptolyngbya* (Zhou et al., 2015), but not for *Plectonema* (Cheng et al., 2017) (Table 3). The latter study, however, did report a shortened latent period, alike we found. Elevated *p*CO_2_ also caused a decrease in latent period for a cyanophage infecting *Synechococcus*, though this was accompanied by a decrease in burst size (Traving et al., 2014). We note that *Synechococcus* is a unicellular marine cyanobacterium, while the other tested cyanobacterial hosts are freshwater filamentous species. Whether the growth strategy and habitat of cyanobacteria play a role in determining the responses of hosts toward phage infections under changing environmental conditions remains to be elucidated. The current lack of data on the effects of *p*CO_2_ on virus proliferation (including viruses infecting eukaryotic hosts), limits further generalizations.

Elevated *p*CO_2_ typically resulted in increased growth rate and biomass build-up of freshwater cyanobacteria species (Verspagen et al., 2014b; Cheng et al., 2017; Shi et al., 2017). As growth rate of hosts likely control phage production (Hadas et al., 1997), it is difficult to separate the direct CO_2_ impact from an indirect effect via CO_2_ induced changes in host growth rate when this is not well controlled [i.e., in batch experiments (Zhou et al., 2015)]. We show that under controlled host growth rates CO_2_ concentration alone could be directly responsible for differences in cyanophage infection and growth characteristics. Higher *p*CO_2_ can partly compensate for the metabolic constraints of P-limitation found to suppress cyanophage production, as long as the limitation is not too severe (i.e., not at the lowest host growth rates of 0.05 d^-1^).

Global environmental and climatic changes, such as shifts in nutrient supply and *p*CO_2_, affect the eco-physiology of cyanobacteria and thereby the formation of blooms (Visser et al., 2016; Huisman et al., 2018). Elevated *p*CO_2_ may promote cyanobacterial blooms, while associated warming may strengthen thermal stratification and subsequently nutrient limitation (O’Neil et al., 2012; De et al., 2013; Verspagen et al., 2014a; Xu et al., 2015). Although anthropogenic nutrient loading facilitates the development of harmful cyanobacterial blooms worldwide (Smith, 2003; Schindler, 2012; Paerl and Otten, 2013), inorganic carbon, light or nutrient limitation eventually causes bloom demise (Huisman et al., 2002; Verspagen et al., 2014b; Paerl et al., 2016). *Phormidium* can form dense blooms (Iwayama et al., 2017) but can also grow well under nutrient limiting conditions (this study; Fujimoto et al., 1997; Singh et al., 2014). Our findings demonstrate a combined effect of *p*CO_2_ and P-limitation on *Phormidium* and its phage, where the impact of elevated *p*CO_2_ on cyanophage PP growth characteristics depends on the extent of P-limitation. As such, the ecological impact of cyanophage infection will differ temporally (before, during, and post bloom) and between lakes with different trophic status.

Under moderate P-limitation, the enhanced cyanophage adsorption and production in combination with higher infective burst size at elevated *p*CO_2_ suggest an enhanced viral control of future *Phormidium* populations. Contrastingly, under severe P-limitation, the consistently lower cyanophage adsorption and EOP and higher abortion with elevated *p*CO_2_ demonstrates that higher CO_2_ levels greatly limit cyanophage PP infections and subsequently reduce viral control of *Phormidium*. In conclusion, we show that the combined effect of reduced P supply and elevated *p*CO_2_ as a result of global change will likely have a severe impact on virus growth characteristics and thereby on the control of harmful cyanobacterial hosts in freshwater ecosystems.

## Author Contributions

KC and TF did all the experiments. CB improved the quality of the manuscript. DVW designed the experiments and improved the manuscript.

## Conflict of Interest Statement

The authors declare that the research was conducted in the absence of any commercial or financial relationships that could be construed as a potential conflict of interest.
