# Supplementary material for: Cyanophage Propagation in the Freshwater Cyanobacterium Phormidium Is Constrained by Phosphorus Limitation and Enhanced by Elevated pCO2
Source: Front Microbiol. 2019 Mar 29;10:617. doi: 10.3389/fmicb.2019.00617 (PMC6449453; doi:10.3389/fmicb.2019.00617)
Supplement: Supplementary file 1 [file Table_1.docx]

Supplementary Material

Cyanophage propagation in the freshwater cyanobacterium Phormidium is constrained by phosphorus limitation and enhanced by elevated *p*CO_2_

Kai Cheng^1,2*^, Thijs Frenken^2^, Corina P. D. Brussaard^3^, Dedmer B. Van de Waal^2^

*** Correspondence:** Kai Cheng chengkaicn@163.com

# Supplementary Table

**Supplementary Table 1.** Overview of interaction of P limitation and elevated *p*CO_2_ to virus infection characteristics..

| Infection characteristics | Interaction | Impact of elevated *p*CO_2_ at different P-limitation conditions | | |
| --- | --- | --- | --- | --- |
|  |  | Severe | Intermediate | Least |
| Infective burst size | * | ↓ | ↑ | ↑ |
| Latent period | NA | - | ↓ | ↓ |
| Infective production rate | * | - | ↑ | ↑ |
| Adsorption ratio | NA | ↓ | ↓ | ↑ |
| EOP | * | ↓ | ↓ | ↑ |
| Abortion percentage | NA | ↑ | ↑ | NA |

Symbols: “*” the interaction between elevated *p*CO_2_ and P-limitation is significant at P<0.001; “-” No impact; “↑” Increase; “↓” Decrease; “NA” Not available.
